# Pure Milk

**Published:** 1897-04

**Authors:** R. A. Pearson

**Affiliations:** Assistant Chief, Dairy Division, United States Department of Agriculture


### THE JOURNAL

OF

### COMPARATIVE MEDICINE AND VETERINARY ARCHIVES.

VOL. XVIII. *APRIL*, 1897. No. 4.

### PURE MILK.

By R. A. PEARSON, B.S.,

ASSISTANT CHIEF, DAIRY DIVISION, UNITED STATES DEPARTMENT OF AGRICULTURE.

THE question of pure foods is one receiving much attention. Those foods having an indefinite composition and whose values are not easily determined by a superficial examination are, naturally, the ones that the strongest efforts are being made to improve. Of these milk is the most important. It is unnecessary at this place to dwell upon the value of milk and its products as food, or to refer at length to the large number of people, especially the sick and the young—the ones least of all able to resist disease—who depend mostly or entirely upon milk for their subsistence. This fluid contains all the constituents of a complete food, and when it is fresh they are in a condition to be easily digested. It is natural that such a medium should be, and is, easily contaminated, and much interest has been aroused in the efforts being made to produce a pure article. Producers appreciate the importance of reform, and methods of producing pure milk are frequently discussed in farmers' meetings.

The purity of milk depends upon the selection, health, care, and feed of the animals; the cleanliness of the utensils; the degree of cleanliness that obtains in the stable and all places where milk is allowed to be; the neatness of the persons who handle the milk; the temperature at which it is held; the age of the milk when it reaches the consumer; and its care after the final delivery. The steps through which this delicate fluid passes might be compared to links of a chain—if any one is imperfect, the entire chain is weak, even though every other link be faultless. From a practical standpoint the subject is pretty well covered by popular lectures and many articles in print.

The medical profession and many scientific bodies are interested in the question of pure milk. They look at the matter from a somewhat different standpoint than the farmer, and usually discuss the questions more from a scientific point of view. The subjects occupying their attention are usually the bacterial infection of milk, its germ content, milk as a carrier of disease, the food-value of milk, etc. Very practical papers are frequently offered. Physicians have many opportunities of seeing and following the results of the use of unwholesome milk; they realize that about one-third of all the deaths are of infants, a large proportion of whom are fed on cow's milk, and this food receives its share of blame for the high mortality. Extremely rash statements are sometimes made, and some persons have become so alarmed that they have given up the use of milk. The "milk scare" seems now to be a thing of the past, and it is to be hoped that it will not again be revived. Some of the agencies that have served to increase the confidence of the consumers are the enforcement of laws relating to the composition of milk, the great improvement in the production and care of milk adopted by many leading dairies, and the milk that is practically perfect in every way now to be found on the market in most cities. The improvement in milk sold in cities and towns has been great. Only a few years ago it was the privilege of any dealer to add as much water to his cans or take as much cream from them as he pleased, and as long as he could make the customer believe the milk was good he was satisfied. The general enforcement of laws against skimming and watering has had a marked effect on improving the quality of milk so far as its chemical composition is concerned.

Pure milk, however, does not signify simply milk having a normal chemical composition. It means freedom from any form of contamination, not the least important of which is bacterial contamination. The sanitary side of dairying needs to be brought into greater prominence, and requires much more attention than it has received.

The contaminations of milk may be divided into two kinds: (1) those affecting the quality from a chemical standpoint, and (2) those affecting the quality from a hygienic standpoint. The first relates chiefly to skimming and watering, by which the quantity and relative amounts of the various constituents are changed; the second to the bacteriological content and the various fermentations that may be taking place. It is sometimes attempted to estimate the losses caused by skimming and watering, and enormous amounts are named; but it is not believed that these nearly equal the losses caused by taints or peculiar changes of the milk, or bad effects of milk due to the organisms which it carries.

Milk is a most excellent medium for the development of bacteria; it contains the constituents necessary for germ-growth, and when it is drawn from the animal the temperature is just right for their rapid increase.

A multitude of forms of bacteria thrive in milk, and their growth and multiplication are marked by changes in the taste, odor, color, and appearance of the fluid. The most common species are those forming lactic acid, and causing the milk to become sour. These act on the milk-sugar, which constitutes about 5 per cent. of milk testing 4 per cent. of butter-fat, and produce lactic acid. Delicate tests have been devised for measuring the amount of acid in milk, and it is easily shown that when carelessly handled it contains two or three times as much acid as when of the same age but carefully handled. It should have less than two-tenths of 1 per cent. acid for household use. Several species of bacteria produce lactic acid; they are the most numerous forms about a dairy, and it is practically impossible to keep them all out of the milk. It was formerly supposed that thunder-storms caused milk to sour, but it has been proven that such is not the case; lactic-acid germs are responsible for sour milk.

Other forms cause the milk to become alkaline, in which condition it may curdle. Others produce various colors and cause the natural color to be tinged more or less deeply with red, blue, or yellow. Slimy milk is a fermentation in which the fluidity is partially lost, and when it is attempted to dip from the container long strings may be formed. A soapy taste is caused by another organism. Some of these germs seem to be harmless, but they are all highly objectionable; and when a dairy becomes affected with those causing peculiar changes, the milk is practically worthless until the trouble ceases.

When a milk emits a peculiar odor it is sometimes due to food the animal has eaten or to the milk standing in an atmosphere impregnated with a strong odor. A familiar example of the former class of taints is the taste of garlic, with which milk is affected in the spring and fall. The fact that milk readily takes up odors if exposed to them is well known to housekeepers, who are careful not to store it near vegetables or in any place containing an objectionable smell. Whether the taint of milk is due to feed the cows have eaten or to bacteria may usually be easily determined as follows: If the odor is strong when the milk is first drawn, and gradually decreases, it is probably due to food eaten by the cows. If it is not at first noticed, but gradually becomes apparent, continually growing worse, it is probably due to bacterial growth.

Pathogenic germs, as a rule, have no peculiar effect on milk; but they are readily carried by it, and outbreaks of disease have been due to milk contaminated by them.

Many methods of purifying milk are practised, but none of them are entirely successful. Dirt may be completely removed by patent strainers, filters, or the separator, but the bad effects of dirt cannot be gotten rid of so easily. These devices may make the milk appear clean, but none of them can take out bacteria or the products of their growth.

Sterilization has been recommended as a means of getting around the difficulty. If sufficient heat is applied the micro-organisms will be killed; but the products of their development up to the time of sterilizing remain; besides this, the taste of the milk is materially changed, making it disagreeable to many people, and it is supposed to be less digestible. The operation of sterilization also requires much labor and expense. Pasteurization requires less heat; it does not kill all the bacteria, and it has less effect on the taste of the milk. Much Pasteurized milk is now sold; but the fact that milk has been Pasteurized does not necessarily mean that it is clean or pure—it simply signifies that the active germs have been destroyed, and the milk (if it has been held at a low temperature), after being Pasteurized, is about the same as it was before being heated. The application of heat makes it possible to keep ordinary milk in good condition much longer than could otherwise be done, and at the same time retain its food value. A chief advantage of heat is the destruction of pathogenic germs that may be in the milk, and this is sufficient to cause some physicians to recommend that all milk not known to have been properly cared for and handled be Pasteurized or sterilized before use.

Preservatives are not here considered, as their use is deprecated by the best authorities.

Strainers, filters, Pasteurizing appliances, and other apparatus for cleaning and purifying milk are for the purpose of bringing it back as nearly as possible to the state of cleanliness in which it is found in the udder of the healthy cow. If it were drawn and handled with sufficient care none of these mechanical aids would be necessary. Bacteria are so abundant in the air about a dairy, however, that it is impracticable to secure milk without some infection; but it has been proven beyond a question that it is practicable and profitable to produce milk having but a few hundred bacteria per cubic centimetre, whereas ordinarily the same volume contains many thousand organisms.

(To be continued.)

